# Knowledge, perception and attitude about Crimean Congo Hemorrhagic Fever (CCHF) among medical and pharmacy students of Pakistan

**DOI:** 10.1186/s12889-018-6248-1

**Published:** 2018-12-03

**Authors:** Ali Ahmed, Maria Tanveer, Muhammad Saqlain, Gul Majid Khan

**Affiliations:** Department of Pharmacy, Quaid-I-Azam University, Islamabad, 45320 Pakistan

**Keywords:** CCHF, Healthcare professionals, Knowledge, Eid-ul-Azha, Pakistan’s

## Abstract

**Background:**

Crimean Congo Hemorrhagic (CCHF) is a deadly tick born disease caused by a virus of genus Nairovirus and is endemic in the Middle East, Asia, Africa, and Eastern areas of Europe. Pakistan is a CCHF endemic country with a constant threat of sporadic outbreaks. Health care workers are more prone to CCHF, hence, it is a prerequisite for members of the healthcare team to stay abreast with current knowledge and display positive attitude and perception. This study assessed the medical and pharmacy students’ preparedness level in terms of CCHF control and management.

**Methods:**

A total of 900 consenting students were selected randomly, who completed a predesigned and validated questionnaire which assessed the participant’s general knowledge, emergency preparedness control and management of CCHF. Data were analyzed by SPSS (IBM SPSS version 21). For data analysis percentages, *P*-value, t-test, the independent sample mean, Whitney U test, Kruskal-Wallis test, Logistic regression, and Spearman correlation were utilized.

**Results:**

Among 900 study respondents, 68% were females and 32% were males, out of which physicians (MBBS) students were 48.4%, and pharmacists students were 51.6%. Majority of the respondents 39.9% were from age group of 22–25 years. Overall 43% healthcare students demonstrated good knowledge about disease causes, transmission, and treatment options. Additionally, 81% of the study participants showed positive attitude, whereas, 69% students demonstrated positive perceptions. The correlation coefficient showed positive correlation between attitude- perception (*r* = 0.268, *p* value = 0.000), knowledge- attitude (*r* = 0.234, *p* value = 0.000) and knowledge- perception (*r* = 0.257, *p* value = 0.000).

**Conclusions:**

Knowledge gaps were observed which is alarming. These gaps were multifactorial and mainly due to lack of knowledge, poor motivation, and old syllabus which needs to be addressed. The study results show that it is crucial to evaluate current curriculum and also showing a dire need of awareness seminars, conferences workshops to highlight and educate about the current endemic disease to future health care professionals.

**Electronic supplementary material:**

The online version of this article (10.1186/s12889-018-6248-1) contains supplementary material, which is available to authorized users.

## Background

**Fig. 1:**
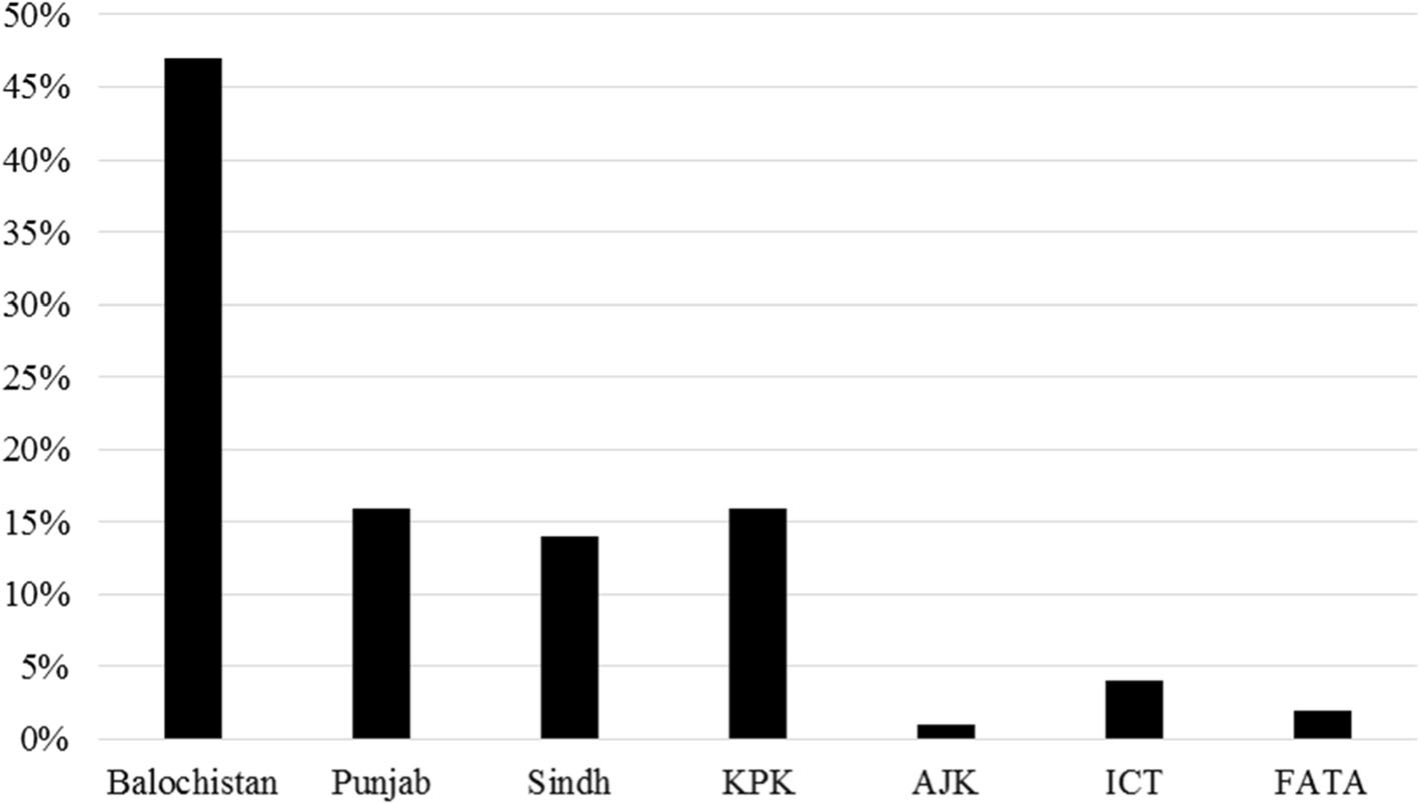
Prevalence of CCHF in Pakistan. Shows the prevalence of CCHF in Pakistan. The highest prevalence is observed in the province of Baluchistan

Crimean Congo Hemorrhagic Fever (CCHF), a zoonotic viral hemorrhagic fever is a cause of significant morbidity and mortality; especially in underdeveloped countries. CCHFV is a member of the genus Nairovirus in the family Bunyaviridae. Basically, this disease is transmitted to humans by domestic animals and bite of an infected tick or via aerosol generated from infected animals’ excreta. Human to human transmission occurs following contact with an infected person’s blood, tissue or fluid discharge [1]. The vectors of this arthropod-borne disease are generally hard ticks of Ixodidae family, including some species of *Rhipicephalus*, *Boophilus*, *Dermacentor* and *Hyalomma* (in particular *Hyalomma marginatum*). Some species of *Argas*and *Ornithodoros* in Argasidae family have been reported to be infected The highly lethal virus is known for producing devastating outbreaks in humans which are very common in areas with developing healthcare systems such as in Africa, Middle East Asia, and Balkans. CCHF outbreaks constitute a threat to public health services because of its prolonged and intense course of infection. It has epidemic potential, high case fatality ratio (10–40%), and difficulties in treatment and prevention. The Hyalomma tick bite infection has a high rate of nosocomial transmission especially due to direct human to human contact [2]. In Pakistan, sporadic outbreaks have been reported frequently, mostly due to contact with viremic livestock blood and nosocomial transmission. The hospital-borne spread has been associated with a lack of, or improper use of personal protective equipment when dealing with infected patients. It mostly occurs during early contact with an undiagnosed patient before taking appropriate protective measures [3]. The first case of CCHF in Pakistan was reported in 1976 and since then continuous cases of CCHF have been emerging throughout the country. In Pakistan 2010, outbreak in Khyber Pakhtunkhwa (KPK) province precipitated and 100 cases were reported and had a 10% fatality rate. Similarly on 11 July 2014, in Hayatabad Medical Complex (HMC), Kpk, 8 patients died, out of which 6 were Afghan nationals and a nurse [4]. Figure 1 Illustrates the prevalence of CCHF, from 2012 to 2016. In accordance with the ministry of the national institute of health, a total of 323 cases were confirmed [5]. Baluchistan remains the most affected province followed by KPK and Punjab. As Pakistan shares, a long border with Afghanistan so large number of patients from Afghanistan come to Pakistani hospitals especially in Peshawar, Quetta, and Islamabad making Congo infection of particular danger in these areas. Figure 1, shows that since the last 5 years about 47% of cases were reported from Baluchistan followed by 17% from Punjab 15% from KPK. Whereas, 14% cases were reported from Sindh followed by 4% from Islamabad and 2% from Federally Administered Tribal Areas (FATA) and lastly 1% from Azad Jammu and Kashmir (AJK) [6].

In Pakistan, the overall environmental cycles are continuously subjected to major changes partly due to the effect of global warming. Such climatic changes affect the initiation and spread of disease outbreaks [7]. A similar trend of CCHF spread has been observed. During extremely hot weather the spread of CCHF experiences, a fall back followed by a subsequent rise during average weather. CCHF is an occupational disease for livestock workers, butchers, slaughterhouse workers, health care workers, animal husbandry workers and veterinarians who belong to a group of people at high risk of the disease in the endemic areas of CCHF [8]. The incidence rate increases by many folds during Eid ul Adha, a religious festival during which millions of animals are slaughtered [6, 9]. As CCHF is a viral contagious endemic disease, cases of spread of this disease in healthcare professionals have also observed. In Quetta, a doctor died due to CCHF [10]. Another doctor died of Congo fever in Abbottabad [11]. A Surgeon and nurse from Bahawalpur hospital died of CCHF at Agha Khan University Hospital (AKUH) in 2016 [12].

Healthcare professionals are a high-risk group and the primary custodians for the provision of health-related treatments. Although education campaigns have increased their awareness regarding CCHF yet it remains unclear to what extent this knowledge can be put into practice and to what extent this practice actually reduces CCHF infection spread. Knowledge, attitude, and perception survey provide a suitable format to evaluate existing programs and to identify effective strategies for behavior change in society [13]. Therefore, it is necessary to evaluate the knowledge, attitude, and perceptions of future medical and pharmacy graduates. The main purpose of this study was to identify the knowledge, attitude, and perceptions of medical and pharmacy students about CCHF so a questionnaire assessing these aspects was designed and validated.

## Methods

### Study design

A cross-sectional study was carried out amongst students of medical and pharmacy government and private universities of Pakistan from August 2016 to January 2017.

### Study location

The study was conducted in Islamabad (capital of Pakistan), Rawalpindi, Lahore, and Faisalabad. Islamabad and Rawalpindi are located in the Potohar region of northern Punjab. The data was collected from government and private medical and pharmacy universities. From Islamabad data was collected from Riphah International University Islamabad, Quaid I Azam University Islamabad, Shaheed Zulfiqar Ali Bhutto Medical University Islamabad. From Rawalpindi data collected from Margalla Institute of Health Sciences and Rawalpindi medical college. From Lahore data was collected from the University of Lahore, Akhtar Saeed Medical and Dental College, Allama Iqbal medical college, Superior University Lahore campus. From Faisalabad data was collected from Punjab Medical College Faisalabad.

### Ethical approval

The ethical approval was obtained from Ethical and Research Board of Department of Pharmacy, Quaid-I-Azam University, Islamabad (Letter No. QAU/pharmacyDept/213). Further approval obtained from ethical and research committee of Pakistan Institute of Medical Sciences, PIMS (Letter No. F.1–1/2015/ERB/SZABMU/08/16) and other Universities and colleges allowed the research to be conducted on the basis of University approval letter.

### Sampling and data collection method

A random sample technique was used to collect data from medical and pharmacy institutions. A self-developed pre-validated questionnaire along with written consent was administered among students at the end of lectures in their classrooms. After describing objective of study and nature of research students were asked to fill questionnaires in presence of the principal investigator. The consenting participants were guaranteed of confidentiality and informed of their right to withdraw from the research at any time when they wanted. Afterward filled questionnaires were collected from students by the investigator and later scoring was done. All poorly filled questionnaires were excluded from the study.

### Questionnaire development

The questionnaire was developed after a thorough review of the literature and the items were evaluated and reviewed for validity by the research committee comprised of senior academic teachers pharmacist and physicians having relevant studies experience. The questionnaire consists of four parts assessing demographics, knowledge, attitude, and perceptions of students. The primary survey was performed a pilot study on 40 participants in order to achieve construct validation and to assess following aspects. All the Cronbach alpha values were above 0.50 (0.565–0.871) which is an indication that the questionnaire is a significantly effective tool for measuring desired objectives and proves statistical validation. Questionnaire is included as Additional file 1.

### Study participants

Study subjects were selected on basis of inclusion and exclusion criteria. According to inclusion criteria participants from4th year and final year MBBS, PharmD, MS/MPhil and Ph.D. students of medical and pharmacy field were selected. Out of 1263 questionnaires were distributed in study participants and 981 (78%) questionnaires were returned. While the questionnaires which were not appropriately filled like missing information were removed finally only 900 (71%) questionnaire were included in the study.

### Data analysis

Data analysis was performed using SPPS version 21.0 (IBM, Armonk, NY, USA). Descriptive statistics (frequencies, median and percentages) were calculated for data analysis. Normality of data is determined by using Kolmogorov–Smirnov test value (*P* < 0.001) as data contain more than 20,000 elements. Non-parametric test was used as inferential statistics tools. Independent sample Mann-Whitney U tests were employed to determine variation in student’s attitude and perceptions regarding Congo fever between gender, study course, marital status and college. Independent-sample Kruskal–Wallis tests were used to assess differences among age groups and study year of students with regard to their attitude and perceptions regarding Congo fever. Furthermore, logistic regression analysis was performed to explore factors responsible of good knowledge regarding Congo fever. Results are expressed as ORs accompanied by 95% CIs, and *P* < 0.05 was used for statistical significance of differences. Pearson correlation test was performed to determine correlation between knowledge, perception and attitude items.

## Results

### Characteristics of participants

Total 900 students were investigated. Most of the participants were female (*n* = 612, 68%) while (*n* = 288, 32%) were males. The proportion of respondents includes 44.9% from 4^th^ year students, 44.4% from 5th year students, 9.3% from MPhil scholars, and 1.3% from PhD scholars. Pharmacy students were over half (*n* = 464, 52.6%) of the total respondents. Most of the surveyed students ware unmarried (*n* = 532, 59.1%) and were from the age group of 22–25 (*n* = 359, 39.9%) Table 1.

**Table 1:** Study population characteristics

Variables	Category	Frequency	Percent
Gender	Female	612	68.0
	Male	288	32.0
Course of Study	Physician (MBBS)	436	48.4
	Pharmacist	464	51.6
Year of Study	PhD	12	1.3
	M.Phil.	84	9.30
	5th year	400	44.4
	4th year	404	44.9
Age	18–21	292	32.4
	22–25	359	39.9
	26 and above	249	27.7
Marital Status	Yes	368	40.9
	No	532	59.1
College	Public Sector	431	47.9
	Private Sector	469	52.1

**Fig. 2:**
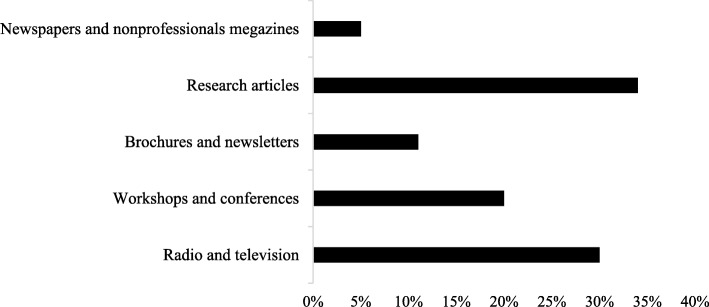
Sources of CCHF. Shows the sources of information used by student healthcare professionals to seek guidance about CCHF

Figure 2, represents sources of information used by students in seeking information about CCHF. The majority of the respondents considered research articles as the major source of information, followed by radio, television, workshops & conferences. Only 11% considered brochures and newsletters to be the best source of information about CCHF.

### Knowledge about CCHF

Table 2, represents the results obtained from the knowledge assessing items of the questionnaire. 24 questions were askedandmixed responses were observed amongst the respondents. More than (79%) of the respondents correctly identified that contact with infected vector leads to the transmission of CCHF, And almost equal proportion (79%) of respondents correctly know about causative agent of CCHF. About more than half of the respondents (59%), correctly identified the best prophylactic measure against CCHF and (35%) gave an incorrect answer to the route of administration of anti-CCHF drug Ribavirin. Additionally, (66%) study participants were also well familiar with the symptoms of CCHF viral disease and similarly (61%) were familiar with standard treatment options available for CCHF. In addition, more than half of the respondents also incorrectly identified that water and air are causative factors for CCHF transmission. On the other hand, 81% of the respondents incorrectly identified the loading dose of ribavirin. About 62% of the respondents were unable to provide a correct answer about the most affected province of Pakistan. When the question was asked about the most affected months of the year only 21% of study participants could answer correctly. About 58% of study participants were well familiar with the diagnostic options available for CCHF. Only 46% of study participants were well aware of the mortality rate of CCHF. Overall from knowledge section results, 43% of the students showed good knowledge, the criteria of which is correct response to 14 questions out of total 24. Among the participants, 58% medical students demonstrated good knowledge while only 28% pharmacy students demonstrated good knowledge Tables 2 and 3.

**Table 2:** Students response to knowledge items

Sr. No.	Question	Response	
		Correct	Incorrect
1	Causing factor of CCHF	712(79.10%)	188(20.88%)
2	spread of CCHF occurs through	541(60.15%)	359(39.88%)
3	Contact with infected vector can be mode of Transmission to human	712(79.10%)	188(20.88%)
4	Contact with infected human blood and body fluids can also be transmission source	747(83.00%)	153(17%)
5	Contact with animals cannot transfer CCHF	431(47.90%)	469(52.11%)
6	Most affected province of Pakistan?	337(37.40%)	563(62.55%)
7	Most affected months of the year?	197(21.90%)	703(78.10%)
8	CCHF can be transmitted through percutaneous contact?	637(70.08%)	263(29.20%)
9	Most common cause of hospital born Congo Infection?	609(67.70%)	291(32.30%)
10	The predominant symptoms associated with CCHF are:	594(66.00%)	306(34.00%)
11	CCHF is highly symptomatic in infected animals:	312(34.70%)	588(65.30%)
12	Mortality rate of CCHF in Pakistan?	415(46.10%)	485(53.90%)
13	What diagnostic option (s) is available for CCHF?	522(58.00%)	378(42.00%)
14	Standard treatment option available for CCHF?	547(60.80%)	353(39.20%)
15	Best prophylactic measure(s) against CCHF?	531(59.00%)	369(41.00%)
16	Is CCHF a zoonotic disease?	647(71.90%)	253(28.10%)
17	Can CCHF be transmitted via air and water?	412(45.80%)	488(54.20%)
18	Can CCHF be transferred through social contacts	333(37.00%)	567(63.00%)
19	Can CCHF be completely cured with medicine?	229(25.40%)	671(74.60%)
20	Contact with feces, urine and saliva of infected person can cause		
	CCHF?	635(70.60%)	265(29.40%)
21	Does avoiding mosquito’s bites prevents CCHF?	418(46.40%)	482(53.60%)
22	Ribavirin is taken as orally?	589(65.40%)	311(34.60%)
23	Loading dose of Ribavirin taken for CCHF is:	170 (18.90%)	730(81.10%)
24	Is there any vaccine available for CCHF?	565(62.80%)	335(37.20%)

Knowledge section was assessed by giving a score of 1 to correct answer and 0 to wrong answer. A score of greater than equal to 14 was regarded as good and a score of less than 14 was regarded as poor

**Table 3:** Variation in respondents’ knowledge by sample characteristics

Variables	Category	Knowledge	
		Good knowledge	Poor knowledge
Gender	Female	277 (45.26%)	335 (54.74%)
	Male	109 (37.97%)	179 (62.03%)
Profession	Physician	254 (58.26%)	182 (41.74%)
	Pharmacist	132 (28.44%)	332 (71.55%)
Age	18–21	87 (29.79%)	205 (70.21%)
	22–25	129 (35.90%)	230 (64.07%)
	26 <	170 (68.27%)	79 (31.73%)
Marital status	Married	236 (64.13%)	132 (35.87%)
	Single	150 (28.20%)	382 (71.80%)
College	Public Sector	220 (51.04%)	211 (48.96%)
	Private Sector	166 (35.39%)	303 (64.61%)
Study Year	Ph.D.	04 (33.30%)	08 (66.67%)
	M.Phil.	54 (64.29%)	30 (35.71%)
	5th year	153 (38.25%)	247 (61.75%)
	4th year	175(43.32%)	229(56.68%)

Knowledge section was assessed by giving a score of 1 to correct answer and 0 to wrong answer. A score of greater than equal to 14 was regarded as good and a score of less than 14 was regarded as poor

### Attitude about CCHF

All respondents responded to all 6 parameters on their attitude of CCHF. From a maximum score of 5 (100%) for positive attitude, respondents obtained a median score of 4 (IQR 1). These results demonstrate that participants have a strongly positive attitude regarding CCHF. About 71% (*n* = 635) participants strongly agreed/agreed that early diagnosis can result in the rapid resolution of symptoms (median 4, IQR 2). Similarly, 67.5% (*n* = 608)participants strongly agreed/agreed that “supportive care helpful for CCHF” (median 4, IQR 2). On the other hand, 71% (*n* = 666) of participants strongly agreed/agreed that “there should be isolated room for CCHF confirmed patient” (median 4, IQR 2), and 66.5% (*n* = 598) of participants strongly agreed/ agreed that “lack of effective isolation state building facilities pose a significant risk to health professionals dealing with infected individuals” (median 4, IQR 2) Table 4.

**Table 4:** Students response to attitude items

Attitude assessing questions	Response					Median (IQR)
	SD(%)	D(%)	N(%)	A(%)	SA(%)	
Effect of early diagnosis on CCHF	62 (6.9)	69(7.7)	134 (14.9)	364(40.9)	271 (30.1)	4(2)
Is supportive care helpful for CCHF	35 (3.9)	72 (8)	185 (20.6)	382 (42.4)	226 (25.1)	4(2)
Are you at risk of contracting CCHF?	31(3.4)	91(10.1)	180 (20)	350 (38.9)	248 (27.6)	4(2)
Do you feel concerned while dealing with infected individuals?	62(6.9)	156(17.3)	221 (24.6)	354 (39.3)	107 (11.9)	4(1)
Is Health care system effectively equipped?	102 (11.3)	313 (34.8)	175 (19.4)	259 (28.8)	51 (5.7)	3(2)
Should there be an isolated room for CCHF confirmed patient?	39(4.3)	84 (9.3)	138(15.3)	352 (36.1)	314 (34.9)	4(2)
Attitude score overall						4(1)

### Perception about CCHF

All participants supplied responses to all seven statements regarding their perception about CCHF. From a maximum score of 5 (100%) for good perceptions, respondents obtained a median score of 4 (IQR 1). Therefore, participants demonstrated good perceptions regarding CCHF.About57.4% (*n* = 517) participants strongly agreed/agreed that use of preventive medicines when dealing with patients suffering from highly contagious diseases is beneficial. 73.2% (*n* = 672) Participants strongly agreed/agreed that all healthcare students and professionals should go for mandatory CCHF testing during sporadic outbreaks(median 4, IQR 1).71.6% (*n* = 645) of participants strongly agreed/agreed that herders of animals, individuals working with livestock and slaughterhouse workers are at a higher risk of CCHF infection (median 4, IQR 1) Table 5.

**Table 5:** Students response to perception items

Perception assessing questions	Response					Median (IQR)
	SD(%)	D(%)	N(%)	A(%)	SA(%)	
Will you follow standard procedures to minimize the risk of transmission of infection?	32(6.6)	67 (14.02)	96 (20.08)	250 (52.3)	33(6.9)	4(1)
Are you equipped will necessary skills to protect yourself while dealing with CCHF patients?	69(7.7)	136(15.1)	185(20.6)	423(47)	87(9.7)	4(1)
Use of preventive medicines while dealing with CCHF patients?	49 (5.4)	159 (17.7)	175 (19.4)	417 (46.3)	100(11.1)	4(1)
You have valuable sources of information for CCHF?	53 (5.9)	152 (16.9)	195 (21.7)	442 (49.1)	58(6.4)	4(1)
All healthcare professionals should go for mandatory CCHF testing during sporadic outbreaks	20 (2.2)	80 (8.9)	128 (14.2)	575 (63.9)	97(10.8)	4(1)
Having pets increases risk of CCHF?	36 (4)	101 (11.2)	190 (21.1)	444 (49.3)	129(14.3)	4(1)
Animal herders are at additional risk of contracting disease	51 (5.7)	73 (8.1)	131 (14.6)	426 (47.3)	219(24.3)	4(1)
Overall Perception score						4(1)

### Differences in student’s knowledge, attitude, and perception

Variation in students’ attitude and perception regarding Congo fever by characteristics were checked. According to independent-sample Mann–Whitney *U* tests, professional degree (MBBS, PharmD) showed statistically significant differences(*P* < 0.05). MBBS students had higher attitude and perception scores (median 4, *P* < 0.001) than Pharmacy students. Those students which are studying in Public universities and colleges had a positive attitude (median 4, *P* < 0.001) than private university students. Married students had more positive attitude and perceptions (median 4, *P* < 0.001) than unmarried students. In independent-sample Kruskal–Wallis tests, we found that there was statistically significant variation between student’s attitude and perception and different age groups (median 4, *P* < 0.001) while study year have statistically significant differences in the only perception of students (median 4, *P* = 0.004) Table 6.

**Table 6:** Variation in Students attitude and perception regarding Congo fever by characteristics

Variables	Category	Attitude			Perception		
		Median (IQR)	Rank	*P*-value	Median	Rank	*P*-Value
Gender^a^	Male	4(1.5)	442.9	0.536	4(1)	440.2	0.345
	Female	4(1.0)	454.1		4(1)	455.4	
Profession^a^	MBBS	4(1)	537.1	< 0.001	4(1)	480.2	< 0.001
	Pharmacist	4(1)	369.1		4(1)	422.6	
Age^b^	18-21	4(1)	370.5	< 0.001	4(1)	397.8	< 0.001
	22–25	4(1)	412.7		4(1)	438.6	
	26 and above	4(1)	598.8		4(0)	529.5	
College^a^	Public	4(1.5)	497.1	< 0.001	4(1)	452.3	0.816
	Private	4(1)	407.7		4(1)	448.8	
Marital status^a^	Married	4(1)	555.6	< 0.001	4(0)	516.3	< 0.001
	Unmarried	4(1)	377.8		4(1)	416.1	
Study year^b^	PhD	4(1)	401.5	0.468	4(1)	425.3	0.004
	M.Phil.	4(0)	460.8		4(0)	535.1	
	5th Year	4(1.5)	462.5		4(1)	437.1	
	4th Year	4(1)	437.9		4(1)	446.2	

^a^Independent sample Mann-Whitney U test

^b^Independent sample kruskall-wallis test

*P* < 0.05 (2-tailed) considered significant

Logistic regression analysis revealed that females (OR 1.45, 95% CI 1.046–2.01; *P* = 0.024), age group 22–25 years (OR 0.482 95% CI 0.324–0.716*P* < 0.001), public sector universities (OR 2.083, 95% CI 1.513–2.868; *P* < 0.001), 5th year students (OR 0.546, 95% CI 0.392–0.762; *P* < 0.001 and married (OR 2.703, 95% CI 1.831–3.989; *P* < 0.001) were the factors associated with the good knowledge regarding CCHF. Table 7.Significant linear positive correlation between attitude-perception (*r* = 0.268, *p* value = .000), knowledge-attitude (*r* = 0.234, *p* value = .000), and knowledge-perception (*r* = 0.257, *p* value = .000) was observed Table 8.

**Table 7:** Logistic regression analysis for factor associated with Good knowledge regarding Congo fever

Variables	Knowledge		Odds ratio	*P*-Value
	Good knowledge	Poor knowledge	(95% CI)	
Gender				
Female	277 (45.26%)	335 (54.74%)	1.45(1.046–2.01)	0.026
Male	109 (37.97%)	179 (62.03%)	Reference	–
Profession				
Physician	254 (58.26%)	182 (41.74%)	1.413(0.974–2.049)	0.068
Pharmacist	132 (28.44%)	332 (71.55%)	Reference	–
Age				
18–21	87 (29.79%)	205 (70.21%)	0.337(0.217–0.523)	< 0.001
22–25	129 (35.90%)	230 (64.07%)	0.482(0.324–0.716)	< 0.001
26 <	170 (68.27%)	79 (31.73%)	Reference	–
Marital status				
Married	236 (64.13%)	132 (35.87%)	2.703(1.831–3.989)	< 0.001
Single	150 (28.20%)	382 (71.80%)	Reference	–
College				
Public Sector	220 (51.04%)	211 (48.96%)	2.083(1.513–2.868)	< 0.001
Private Sector	166 (35.39%)	303 (64.61%)	Reference	–
Study Year				
Ph.D	04 (33.30%)	08 (66.67%)	0.320(0.084–1.220)	0.095
M.Phil	54 (64.29%)	30 (35.71%)	1.389(0.808–2.389)	0.235
5th year	153 (38.25%)	247 (61.75%)	0.546(0.392–0.762)	< 0.001
4th year	175(43.32%)	229(56.68%)	Reference	

Knowledge section was assessed by giving a score of 1 to correct answer and 0 to wrong answer. A score of greater than equal to 14 was regarded as good and a score of less than 14 was regarded as poor

OR = Odds Ratio

*P* < 0.05 (2-tailed) considered significant

**Table 8:** Correlation between scores of knowledge, attitude, and perception

Variable	Correlation Coefficient	*P*-Value
Attitude-Perception	0.268^a^	.000
Knowledge-Attitude	0.234^a^	.000
Knowledge-Perception	0.257^a^	.000

^a^Correlation significant at 0.01 level (2 tailed)

## Discussion

To the best of our knowledge, there is no reported study that has evaluated thoroughly knowledge, attitude, and perceptions of medical and pharmacy students about CCHF in Pakistan. In order to effectively deal with CCHF patients with minimum risks, healthcare professionals need to have good knowledge about precautionary measures like wearing of gloves, masks, protective clothing, goggles, disposables gowns and face shields before visiting patients. Transmission can occur through direct contact with infected blood, needle stick injuries, and contact with infected abraded skin and during disposal of infected waste. In Pakistan, a qualified general surgeon died of CCHF while dealing with CCHF patient due to nosocomial transmission. Many other examples of this type can be observed frequently throughout the world [12].

The previous such study conducted in students of a single college in Pakistan concluded that students have poor knowledge even about cause and source of transmission of CHF because respondents were nonhealthcare students like a business and social science students. The results of our study are batter to this study as the participants in our case are related to health care students [14]. When the results of our study were compared with the study conducted in healthcare professionals of Iran, similar results were found. But Iranian study demonstrated more knowledge of participants [15]. According to Iranian study when question CCHF can be transmitted through percutaneous contact from an infected individual was asked, 89.5% provided a correct answer while in our study 71% participants provided the correct answer. Similarly, in Turkish study 98.2% healthcare provided a correct answer to this question [15, 16]. Furthermore, in our study, 53% of participants provided the wrong answer about vaccine availability. These results are confirmed with the similar study conducted previously in healthcare professionals of Baluchistan, Pakistan [17]. Baluchistan study also highlighted the major lacks of knowledge, especially in preventive and burial procedures. Knowledge about the causes of disease was good but major deficiency was observed in transmission, epidemiology, and treatment of disease. Table 3, overall female healthcare students have more knowledge then male participants. The reason might be the hard work and more interest of female students in health care practices. Knowledge of healthcare students increased with age and married students are quite good in this as they might attend number of clinical rounds, conferences, and workshops. so they have more knowledge about the disease [18]. Medical students knowledge was significantly better than that of pharmacist students [19]. This could be possibly explained by the current health care system in Pakistan where medical students are seen as more clinically oriented professionals than other professionals because of their in-depth clinical training and more opportunities for professional development. However, it is equally important to educate Pharmacist, as they are at equal risk of acquiring and transmitting infections such as CCHF. There is a need to encourage these workers to educate themselves with an updated knowledge of infections and other healthcare issues by participating in educational and related programs.

More than 70% healthcare professionals favored there should be an isolated room for the CCHF confirmed patients. These results are consistent with the studies previously done in healthcare professionals in Iran. These results were also consistent with the results of a study conducted in rural Georgian village [20]. However, in one question highly negative attitude was observed like commenting on the statement ‘is healthcare system of Pakistan is effectively equipped with the treatment facilities or not’ only about 34% participants agreed with the statement while the majority of participants opposed the statement with a negative attitude. This result is consistent with these studies [16, 21]. Findings of these results revealed that with huge positive attitude if healthcare professionals equip themselves with the basic knowledge of CCHF disease, better treatment of patients can be done with limited resources.

Among the participants 59% agreed on to follow standard procedures to minimize the risk of infections,57% students agreed that they are effectively equipped with necessary observing skills, 57% participants agreed with the use of preventive medicine and 56% showed positive response about whether they have valuable sources of information. These results are somewhat similar to the studies conducted previously in different countries [20, 22]. Majority of participants agreed that animal herders and pets increased the risk of CCHF infection just like Turkish study [23]. The mean attitude and perceptions of the participants were positive. The attitudes and perceptions of the physician students were more positive than that of pharmacist students. This finding could be interpreted to mean that physician students are more aware of their patient’s clinical condition and are responsible for counseling patients, which reflects positivity in their attitudes and perceptions about the disease.

Although the national institute of Health (NIH), Pakistan has already advised that healthcare professionals are at increased risk of contracting the disease. Despite the welcoming and positive attitude and perceptions of students towards CCHF, knowledge of students about CCHF was very poor. Possible reasons include lack of educational programs necessarily dealing the outbreak of disease.

### Strengths and limitations of the study

The strength of this study is that it has focused on the area where not much literature is available from Pakistan. The results of this study can help the stakeholders and other health officials to evaluate the effectiveness of their policies about CCHF. The large sample size and the fact that a fairly high proportion of the total number of students from private (52.1%) and public (47.9%) colleges from the capital city and Punjab province are included to strengthen the validity of the research. However, since Pakistan is a country with huge regional differences in socioeconomic and health status, our results may not be representative of all medical and pharmacy students in the country. The reliability and validity of the survey are strengthened through the previous testing of the instrument. However, we cannot ignore the tendency of participants to provide more socially desirable responses.

## Conclusion

The current study has its implications in relation to policy and practice as it provides a current snapshot of the knowledge, attitude, and perception about a life-threatening zoonotic endemic among future health care professionals. The findings of this study indicate that the student HCWs’ knowledge about CCHF was not optimal; however, their attitudes about CCHF were positive. Future studies should be conducted nationwide to validate these results. Interventions should be customized to target areas where participants showed a lack of knowledge and negative attitudes. Study results demonstrate that it is crucial to evaluate current curriculum and there is dire need of awareness seminars, conferences, and workshops about current endemic diseases to future health care professionals.

## Additional file

Additional file 1: Questionnaire used in study. (DOCX 20 kb)

## Abbreviations

AJK: Azad Jammu and Kashmir
AKUH: Agha Khan University Hospital
CCHF: Crimean Congo Hemorrhagic Fever
CCHFV: Crimean Congo Hemorrhagic Fever Virus
FATA: Federally Administered Tribal Areas
HCWs: Healthcare Workers
KPK: Khyber Pakhtunkhwa
