# Supplementary material for: Knowledge, perception and attitude about Crimean Congo Hemorrhagic Fever (CCHF) among medical and pharmacy students of Pakistan
Source: BMC Public Health. 2018 Dec 3;18:1333. doi: 10.1186/s12889-018-6248-1 (PMC6276267; doi:10.1186/s12889-018-6248-1)
Supplement: Supplementary file 1 — Questionnaire used in study. (DOCX 20 kb) [file 12889_2018_6248_MOESM1_ESM.docx]

**Tool for data collection**

**Demographics Questions:**

1. Name:

2. Gender: F ☐ M ☐

3. Age (Years): 18-22 ☐ 22-25 ☐ 26 and above ☐

4. Course of study: Physician (MBBS) ☐ Pharmacist ☐

5. Year of study: PhD ☐ M.Phil ☐ 5^th^ year ☐ 4^th^ year ☐

6. College: Public Sector ☐ Private Sector ☐

8. Marital status: Yes ☐ No ☐

**Knowledge indicator questions:**

1. What you know about the causing factor of CCHF?

Fungi ☐ Bacteria ☐ Virus ☐ No Idea ☐

2. The spread of CCHF occurs through:

Mosquito☐ Ixodid hard ticks☐ Cat fleas☐ Infected flies☐

3. Contact with infected vector can be mode of Transmission to human:

Yes☐ No☐ No Idea ☐

4. Contact with infected human blood and body fluids can also be transmission source:

Yes☐ No☐ No Idea ☐

5. Contact with animals cannot transfer CCHF:

Yes☐ No☐ No Idea ☐

6. Most affected province of Pakistan?

Punjab ☐ Sindh ☐ Balochistan☐ KPK ☐ No Idea ☐

7. Most affected months of the year?

Jan-Mar ☐ Jun-Aug ☐ Sept-Nov ☐

Same throughout the year ☐ No Idea ☐

8. CCHF can be transmitted through percutaneous contact?

Yes☐ No☐ No Idea ☐

9. Most common cause of hospital born Congo infection?

Use of unsterilized medicalequipment☐

Percutaneous infection to laboratory personnel ☐

Poor infection control practices ☐ All of the above ☐

10. The predominant symptoms associated with CCHF are:

Hemorrhage and fever☐ Headache and Joint pain☐

Generalized red spots☐ all of the above☐

11. CCHF is highly symptomatic in infected animals:

Yes☐ No☐ No Idea ☐

12. Mortality rate of CCHF in Pakistan?

Extremely high fatality rate (40-60% ☐ High Fatality rate (10-40%) ☐

Medium Fatality rate (10-20%) ☐ Low Fatality rate (5%) ☐

13. What diagnostic option (s) is available for CCHF?

ELISA (Enzyme-linked Immunosorbent assay)☐

RT-PCR (real time polymerase chain reaction)☐

Combination of ELISA and RT-PCR ☐All of the above ☐

14. Select the standard treatment option available for CCHF?

Ribavirin ☐Fidaxomicin ☐Ceftaroline ☐

Caspofungin ☐No Idea ☐

15. Select the best prophylactic measure(s) against CCHF among the following?

Using DEET insect repellent on exposed skin and clothing ☐

Burning the dead bodies of infected animals ☐

Proper disposal of infectious human blood from hospitals☐

All of the above ☐

16. Is CCHF a zoonotic disease?

Yes ☐ No ☐ No Idea ☐

17. Can CCHF be transmitted via air and water?

Yes ☐ No ☐ No Idea ☐

18. Can CCHF be transferred through social contacts like sharing clothes, Cups/ plates/ spoon/ glass, bathrooms, shaking hands & kissing?

Yes ☐ No ☐ No Idea ☐

19. Can CCHF be completely cured with medicine?

Yes ☐ No ☐ No Idea ☐

20. Contact with feces, urine and saliva of infected person can cause CCHF?

Yes ☐ No ☐ No Idea ☐

21. Does avoiding mosquito’s bites prevents CCHF?

Yes ☐ No ☐ No Idea ☐

22. Ribavirin is taken as orally?

Yes ☐ No ☐ No Idea ☐

23. Loading dose of Ribavirin taken for CCHF is:

2g☐ 4g☐ 6g☐ No Idea ☐

24. Is there any vaccine available for CCHF?

Yes ☐ No ☐ No Idea ☐

**Attitude indicators questions:**

1. Do you think early diagnosis can lead to rapid resolution of symptoms of hemorrhagic fever?

Strongly Disagree☐ Disagree ☐ Neutral ☐Agree ☐ strongly agree ☐

2. Do you think the severity ofdisease can be decrease through the management of electrolyte and fluid imbalance and provision of supportive care through blood, plasma and platelet replacement?

Strongly Disagree☐ Disagree ☐ Neutral ☐ Agree ☐ strongly agree ☐

3. Do you think the lack of effective isolated building facilities pose a significant risk to health professionals dealing with infected individuals?

Strongly Disagree☐ Disagree ☐ Neutral ☐Agree ☐ strongly agree ☐

4. Keeping in view the contagious nature of the infectious agent, do you think you will feel concerned in dealing with infected individuals?

Strongly Disagree☐ Disagree ☐ Neutral ☐ Agree ☐strongly agree ☐

5. Do you think the health care system is effectively equipped to provide isolated body fluid collection and testing setup for Congo infected individuals?

Strongly Disagree☐ Disagree ☐ Neutral ☐ Agree ☐strongly agree ☐

6. Do you think there should be isolated room for CCHF confirmed patient?

Strongly Disagree☐ Disagree ☐ Neutral ☐ Agree ☐ strongly agree ☐

**Perceptions assessing questions:**

1. Do you take standard blood testing procedures to minimize the risk of transmission of infection?

Strongly Disagree ☐Disagree ☐ Neutral ☐ Agree ☐ strongly agree ☐

2. Are you equipped with isolation observing skills necessary to protect yourself from contracting the disease whilst working with infected individuals?

Strongly Disagree☐ Disagree ☐ Neutral ☐ Agree ☐ strongly agree ☐

3. Do you use preventive medicines when dealing with patients suffering from highly contagious diseases?

Strongly Disagree☐ Disagree ☐ Neutral ☐ Agree ☐ strongly agree ☐

4. Do you consider you have a valuable source of information to look up to in case of confusion in relation to dealing with infected individuals?

Strongly Disagree☐ Disagree ☐ Neutral ☐ Agree ☐ strongly agree ☐

5. In my opinion all healthcare students and professionals should go for mandatory CCHF testing during extreme outbreaks:

Strongly Disagree ☐ Disagree☐ Neutral ☐ Agree ☐ strongly agree ☐

6. Do you think having birds and animals at your home can put you at an additional risk for CCHF?

Strongly Disagree☐ Disagree ☐ Neutral ☐Agree ☐ strongly agree ☐

7. Herders of animals, individuals working with livestock and slaughterhouse workers are at a higher risk of CCHF infection?

Strongly Disagree☐ Disagree ☐ Neutral ☐ Agree ☐strongly agree ☐

**Signature..................**
